# Climate change, evolution, and reproductive health: The impact of water insecurity and heat stress on pregnancy and lactation

**DOI:** 10.1093/emph/eoaf008

**Published:** 2025-04-24

**Authors:** Michaela Howells, Aunchalee E L Palmquist, Chloe Josefson, Kelsey Dancause, Elizabeth Quinn, Lukas Daniels, Alexandra Faith Ortiz Blair

**Affiliations:** Department of Anthropology and Geography, Colorado State University Fort Collins, Colorado, USA; Duke Global Health Institute, Duke University, Durham, USA; Department of Biological and Biomedical Sciences, North Carolina Central University, Durham, USA; Département des sciences de l’activité physique, University of Quebec in Montreal, Montreal, Canada; Department of Anthropology, Washington University in St Louis, St. Louis, USA; Department of Anthropology, Washington University in St Louis, St. Louis, USA; University of North Carolina Wilmington, Department of Biology and Marine Biology, Wilmington, NC, USA

**Keywords:** disasters, climate change, reproduction, stress, extreme heat, water insecurity, vulnerability

## Abstract

Global water insecurity and rising heat indices have a significant impact on human health. There is an urgent need to understand these climate impacts on the most physiologically and socially vulnerable populations across the globe and use this information to strengthen evidence-based responses. Pregnancy, postpartum, and the first year of life are especially sensitive to water insecurity and extreme heat exposures, as these stages require significantly more access to hydration and cooling resources than other life stages. Extreme heat and water insecurity are ecological stressors forcing parents and alloparents to make difficult decisions between optimal practices for survival and reducing human suffering. Additionally, these stressors may impose physiological trade-offs at the cost of reproductive performance. Here, we examine the changing effects of water insecurity and heat stress throughout pregnancy and lactation using an interdisciplinary, evolutionary, and biocultural lens. We highlight the importance of an evolutionary medicine framework in efforts to investigate the effects of climate change on global health equity. In addition, we outline implications for public health emphasizing the need for targeted policies and healthcare strategies to support pregnant individuals and lactating individuals in affected regions. By integrating evolutionary perspectives with global health concerns, this paper aims to inform future research agendas and policy frameworks aimed at enhancing resilience and adaptation among populations facing escalating climate challenges during critical reproductive phases.

## HEAT STRESS, WATER SCARCITY, AND VULNERABLE POPULATIONS

Humans are among the most adaptable species on Earth due to the combination of biological and cultural adaptations. However, rapidly increasing ambient temperatures and water scarcity related to a changing climate pose a significant threat to human health and reproduction. In 2024, the summer heat index for both hemispheres exceeded all previous records [1]. Even though the last decade has experienced the 10 hottest years in the National Oceanic and Atmospheric Administration’s (NOAA) 174-year record, each summer is predicted to be the coolest we will experience for the rest of our lives [1, 2]. Who is most at risk, and how can evolutionary perspectives add value to the science of climate change and global health?

A core limit in humans’ ability to respond to changing climate conditions—especially increasing ambient temperatures—is water availability. The average daily water intake for men is 3.98L and 2.7L for nonpregnant, nonlactating women [3]. Individual factors such as physical activity levels, metabolism, and life history stage can significantly influence hydration needs [4–8]. Beyond hydration, water is needed for a range of daily activities and personal use. These include food preparation, hygiene, sanitation, cultural practices, and coping with high ambient temperatures [9]. Water insecurity can lead to significant modifications or restrictions of these behaviors. Although we recognize these additional requirements and potential strains, we focus this review on one of the key biological limiting factors associated with water insecurity—hydration status of pregnant and lactating individuals.

Climate change, when combined with increased demands for water associated with population growth, agricultural and industrial requirements and the rampant mismanagement of water resources and pollution, have heavily contributed to a global water crisis [10]. According to the 2024 United Nations World Water Report [11], approximately half of the world’s population experience water insecurity at some point during a given year. Water insecurity can be driven by absence of water as well as lack of potable water due to contaminated or polluted water sources. Roughly two billion people live in a country that is experiencing water stress with people living in low-income countries, fragile states, and resource constrained environments experience chronic and severe levels of water insecurity. A report released in 2022 noted that an estimated 2.2 billion people did not have access to safely managed drinking water, with increased challenges for those rural populations with weak infrastructures that are unable to manage water and sanitation services.

Individuals who identify as women and girls and those who are assigned female at birth are uniquely affected by water insecurity. Roughly 800,000 women face illness and death associated with lack of access to safe water, sanitation, and hygiene. Indigenous women, refugee women, women with disabilities, and women in economically disadvantaged and socially marginalized groups are among the most vulnerable to water insecurity due to climate change [12]. One report estimates that for 2023, approximately 380 million women and girls across 26 countries were exposed to high or critical water shortages. This same report estimates that the number of women and girls who are affected will rise to 471 million by 2030 across 29 countries [12].

## REPRODUCTIVE TRADE-OFFS SURROUNDING WATER AND HEAT STRESS

Human reproductive processes have shaped our physiology and life history on this planet [13]. However, it was only recently that pregnant people and neonates were identified as particularly vulnerable to environmental challenges like extreme heat [14]. A female-centered biological perspective is essential to understanding the intersection of climate change and reproduction, especially at the mechanistic level, and their impact on physiological trade-offs occurring during the peripartum period [13, 15–18].

Trade-offs are a central tenet within evolutionary medicine and have been investigated as both proximate and ultimate explanations for species’ life history patterns, including reproduction [18–21]. Garland et al. (2022) describe six main categories of trade-offs as they relate to proximate-ultimate causations (Box 1). Hyperthermia and dehydration impose resource limitations on populations that may lead to trade-offs among traits and/or physiological processes. Pregnancy and lactation are unique physiological states that increase energetic and nutritive demands, thereby increasing resource requirements. Hyperthermic temperatures also increase total energy expenditure, as organismal responses to heat (e.g. cutaneous vasodilation, sweating) require energy and act to prevent further increases to core body temperature [26].

Box 1.  **Trade-offs during pregnancy and lactation**

Trade-offs provide a valuable framework to understand adaptations and the evolution of life histories and are of broad interest to many scholars of evolutionary medicine [13]. Though many different definitions of trade-offs exist, we use the simplest definition of trade-offs: when one trait *cannot* (not does not) increase without a decrease in another [23]. Garland et al. (2022) describe six main categories of trade-offs, which are caused by both proximate and ultimate factors. Within the context of pregnancy and lactation, proximate causes are those that underlie the immediate mechanisms behind reproductive performance (e.g. offspring survival and growth, maternal health during these stages, or quality and quantity of milk produced), whereas ultimate causes refer to evolutionary processes that have shaped the traits associated with pregnancy and gestation (e.g. species-typical gestation and lactation patterns, etc.). Generally, the types of trade-offs associated with proximate causes occur at the organismal level and those with ultimate causes may occur over generations, though it is important to note that this distinction is not mutually exclusive, especially in the case of trade-offs related to antagonistic pleiotropy, which has both proximate and ultimate explanations (*cf* [23].). Similarly, trade-offs among variables of interest may fit into multiple categories. In the present paper, we focus on those occurring at the organismal level during reproductive stages, though we acknowledge that climate change impacts every level of biological organization, and therefore, may impact each category of trade-offs described below.

Proximate:

1. Allocation constraints –when there is a limit for a given resource (e.g. macronutrient, energy, time, space, etc.) and partitioning that resource to support one trait results in a decrease in resources available to be allocated towards another
2. Functional conflicts (or constraints)—‘when features that enhance performance of one task decrease performance of another’ [22]
3. Shared biochemical pathways—when control over different traits and/or physiological processes share common molecules, called integrator molecules [24, 25] and/or mediating pathways
4. Antagonistic pleiotropy* - negative additive genetic correlations caused by genetic variants (alleles) that increase one component of fitness while decreasing another; can occur as a result of both proximate and ultimate factors and places proximate causations of trade-offs within the context of genetics [22]

Ultimate:

5. Ecological circumstances (selective regime)—when the presence of trade-offs is context-dependent and depends on the environment in which the organism exists; within the context of climate change, this may mean that limitations on water and/or factors related to metabolism (resulting from hyperthermic conditions) may reveal trade-offs that may otherwise not be present in other populations who do not face the same resource limitations
6. Sexual versus natural selection—when sexually-selected characteristics (i.e. secondary sexual characteristics) increase reproductive success and/or performance at the expense of other traits related to fitness

At the cellular level, biochemical metabolic processes are sped up due to increased kinetic energy, thereby imposing further energy demands [26]. Together, increased demands imposed by hyperthermia may reduce resources available for reproduction. High ambient temperatures may further constrain total energy expenditure, as the ability to dissipate heat generated by metabolic processes is thought to act as an upper limit for metabolic rate in endotherms [27] and may impact patterns of physical activity [28].

**Figure 1.**
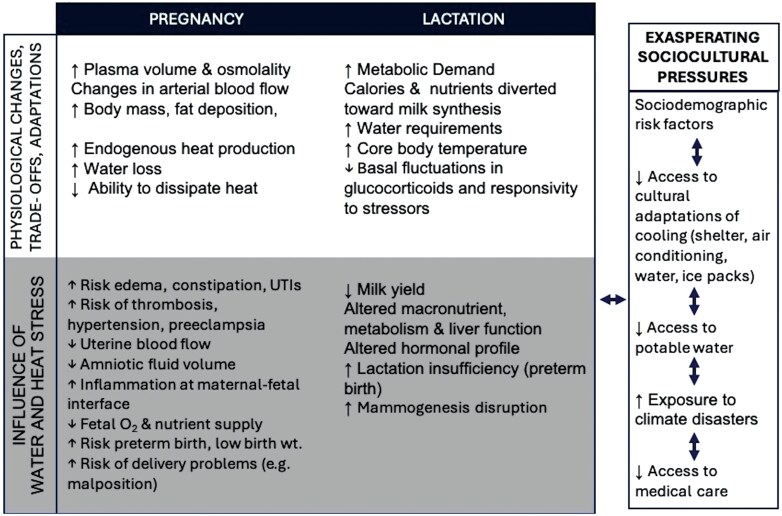
The impact of water stress, heat stress, and exasperating sociocultural factors ontypical physiological changes in pregnancy and lactation.

Human water needs also shift across the lifespan [29]. Perhaps the most pronounced shift in hydration needs emerges for those who are pregnant, birthing, or lactating (Fig. 1). Although everyone needs water to survive, the rapidly changing physiology of individuals in the perinatal period (pregnancy and the first year of life) reflects a particularly emergent need. Similarly, hydration needs during the so-called first 1000 days—from conception to age 2—will vary significantly and have important consequences on development during this time period. This period of development is critically important in evolutionary terms for shaping the bodies and brains of offspring [13].

The majority of studies rely on self-reported data, however, Yamada et al. (2022) employed doubly-labeled water to more accurately measure water turnover. They found a curvilinear relationships between water turnover and air temperature and effective latitude across human populations. Water turnover was increased at higher air temperatures that were associated with hyperthermic conditions. Additionally, their data found that equatorial populations have high water turnover and that water turnover generally decreases with absolute distance from the equator (until 50° effective latitude). Together, these data suggest that the increased water needs during pregnancy and lactation may be further exacerbated by high ambient temperatures and living in locations that are particularly vulnerable to the effects of climate change (e.g. the tropics). Here, we discuss adaptations and limitations related to water insecurity and heat stress surrounding the shifting physiology of pregnancy, parturition, and lactation.

### Pregnancy and water needs

Pregnancy triggers some of the most extreme physiological modifications of water needs over the human lifespan due to thermoregulatory and physiological modifications. As a result, environmental stressors, such as heatwaves and drought, can have acute and chronic effects on immediate, life course, and intergenerational health outcomes [30, 31]. Hydration needs during pregnancy rise due to an increase in blood volume, development and maintenance of amniotic fluid, and maintenance of maternal blood pressure and uteroplacental perfusion. During pregnancy, individuals experience a 45% (~1,200–1,600 mL) increase in blood volume. As such, daily drinking water needs increase by an average of 300 mL [3, 4, 32].

First trimester hydration needs remain similar to pre-pregnancy levels. However, 0.3-3% of pregnant people experience increased urination and/or nausea and vomiting (hyperemesis gravidarum in extreme cases) [33, 34] and may require additional hydration beyond the baseline [35].

Although early pregnancy does not require as substantial an increase in fluids as later stages of pregnancy, it is still a critical time of exposure and disruption for the developing fetus. For instance, hydration restrictions during the first trimester increase the susceptibility of stillbirths more than in any other trimester [36, 37].

The second and third trimesters have significantly greater hydration requirements due to further increases in maternal blood volume and amnionic fluid. These hydration transitions support kidney function and ensure proper circulation [35]. The third trimester has the most extreme increase in fluid requirements to support rising plasma volume. Increased plasma volume helps maintain and support the increased blood volume, blood pressure, and uteroplacental perfusion associated with this stage [38]. Changes in arterial blood flow triggers increased retention of water and sodium in the kidneys [35]. By the later part of the third trimester, plasma volume increases by ~50–60%. Red blood cells do not keep pace with plasma volume increases, reducing plasma osmolality [35]. In short, dehydration during pregnancy disturbs homeostasis and can have a significant effect on pregnancy outcomes.

Dehydration during pregnancy is associated with maternal health complications and adverse outcomes. For instance, dehydration during the third trimester can lead to edema, constipation, and urinary tract infections [39]. Furthermore, reduced vascular volume following dehydration causes reduced uterine blood flow that may trigger release of prostaglandins, thereby increasing the risk of preterm birth [40]. Water-sparing mechanisms, like the release of antiduretic hormone from the posterior pituitary gland might provoke simultaneous release of oxytocin, which could also trigger preterm labor [40]. Dehydration in pregnancy is associated with reduced amniotic fluid volume [41] which could be a risk factor for umbilical cord compression, malpresentation, or malposition [42], although more studies of clinically relevant maternal and fetal outcomes are needed.

### Pregnancy and heat stress

The physiological changes associated with pregnancy require thermoregulatory mechanisms to maintain normal core body temperatures. Increased body mass, fat deposition, and endogenous heat production due to fetal and placental metabolism impedes the ability to dissipate heat and are compensated for by changes such as a lower sweating threshold and an increase in plasma volume and cutaneous circulation flow [40]. Hyperactive adrenal and thyroid functions also result in greater water loss through sweating [43]. Conditions such as high humidity in hot environments restrict evaporative cooling mechanisms that might interfere with these adaptive mechanisms, although whether risk is higher for pregnant than non-pregnant individuals is unclear [40].

Pregnancy also modifies maternal blood composition and flow, and heat exposure likely exacerbates these changes. For example, heat strain activates coagulation pathways in non-pregnant adults. Pregnancy is already a state of hypercoagulability, and further activation of coagulation pathways due to heat stress among pregnant people might increase risk of thrombosis [40]. Observational studies and meta-analyses demonstrate that extreme heat exposure is associated with risk of gestational hypertension and pre-eclampsia [44]. Increased heat exposure during pregnancy is also associated with worsened cardiovascular and respiratory health, psychiatric issues, unfavorable anthropometric outcomes, and overall mortality [45].

Most evidence on physiological mechanisms that might mediate effects of high ambient temperature on pregnancy outcomes is derived from animal models [40, 46]), and more research in diverse populations is needed. Recent observational cohort studies among pregnant subsistence farmers in The Gambia demonstrated clear linear relationships between maternal heat stress and heat strain (based on measures of skin temperature, tympanic temperature, and heart rate) and between maternal heat stress or heat strain with fetal strain (based on fetal heart rate and umbilical artery resistance) [46]. The authors highlighted that although maternal heat strain represents one mechanism linking maternal heat stress to fetal stress, other biological mechanisms need further exploration. Notably, they also highlighted the paucity of field studies testing physiological pathways linking maternal heat stress with maternal and fetal outcomes.

#### Heat stress on placental structure and function

The placenta plays multiple roles in protecting fetal development. It mediates materials passed from maternal to fetal circulation and is involved in fetal respiration, excretory functions, nutrition, hormone production, and immune protection [47]. Changes in this organ’s development and function increases the risk of poor fetal growth, preterm birth and other complications associated with fetal and neonatal morbidity and mortality [48] as well as life-long consequences that impair the health of the fetus and later the neonate [49].

Heat stress challenges the functionality of the placenta. Chronic heat stress can lead to reduced placental growth and impaired fetal oxygen and nutrient supply in animals [50]. Additionally, studies in both animals [51, 52] and humans [53] have found associations between heat stress and placental characteristics, such as placental weight and volume as described below. Maternal heat stress has also been associated with changes in gene expression in the placenta of Saanen goats [54].

Heat exposure affects maternal-fetal blood flow and placental characteristics via multiple pathways. Heat exposure can cause reduced uterine blood flow [55], as increased peripheral flow in response to heat exposure results in reduced placental perfusion. This could compromise nutrient and oxygen delivery to the fetus and, in the longer term, result in decreased placental weight [40, 56]. Furthermore, heat strain causes oxidative stress that results in the release of stress hormones, endotoxins, cytokines, and other inflammatory markers [40].

The inflammatory cascade associated with heat stress could promote inflammation and the release of proinflammatory cytokines and prostaglandins at the maternal-fetal interface [40, 57], potentially triggering uterine contractions and preterm labor [58]. Inflammation and oxidative stress increase blood viscosity following heat exposure, thereby decreasing uterine blood fluidity [40]. Prolonged heat exposure may also promote the release of oxytocin and anti-diuretic hormone. These hormones reduce uterine blood flow and trigger a fetal metabolic change from catabolic to anabolic pathways [40].

### Heat stress and impacts on parturition and neonatal outcomes

Both short-term and long-term exposure to extreme heat can contribute to adverse birth outcomes. Heatwaves and increasing temperatures have been connected with increased risk of premature rupture of membranes (PROM) [37], gestational cardiovascular events during delivery [59], fetal distress [60], shorter gestation lengths or risk of preterm birth [37, 57], placental abruption [61], stillbirth [37, 61], increased risk of neonatal mortality [44], birth defects [44], and low birthweights [44, 62]. Associations between high temperatures and preterm birth are more prevalent in those with chronic diseases [63].

As noted above, risk varies over the course of pregnancy. Data among pregnant subsistence farmers in The Gambia suggest that physiological indicators of heat strain are particularly influenced by environmental conditions in the third trimester of pregnancy compared to the second [64]. A meta-analysis involving 70 studies across 27 countries identified multiple periods throughout pregnancy vulnerable to high temperatures. These include the month prior to conception, the month of conception, the first and second trimesters, and the final week of pregnancy [37]. Maternal hydration status, likely mediated through the placenta, may play a role in these relationships. For example, prospective cohort studies among 38 pregnant participants in Indonesia showed that dehydration from 32 to 37 weeks of pregnancy was associated with smaller birth weight, length, chest circumference, and head circumferences [65].

Models from meta-analyses and cohort studies demonstrate marked predicted effects of temperature increases on adverse birth outcomes. For example, Chersich et al. (2020) found that the risk of preterm birth and stillbirth increases 5% with each additional degree Fahrenheit (0.56°C). The increased risk of preterm birth was 16%, and 46% for stillbirths [53] on heatwave compared to non-heatwave days. Similarly, studies among 7,585 pregnant participants in Spain suggest an estimated 5-day reduction in average gestational age at delivery following days with unusually high heat–humidity index [57]. Longer-term changes in temperature have also been associated with birth outcomes. Models of data 30-year temperature trends and birthweights from 63 countries suggest that birthweight will decrease by 0.44–1.05% per °C increase in temperature under projected climate change [62].

### Lactation and water needs

Successful lactation requires energy to support processes independent of energy exported as milk, such as those related to mammary development and maintenance of secretory activity. As such, it is hypothesized that individual variation in lactation (i.e. the energetic density and quantity of milk produced) may be due, at least in part, to individual variation in resource acquisition—including hydration and metabolic factors [15, 66–68]. Further, the high demands of reproduction, and in particular lactation, are often assumed to result in trade-offs in energy allocation [15, 69–71].

Changes in fluid balance, either resulting from decreased water intake or increased output via sweating, also impacts lactation and may further compound the effects of increased ambient temperatures. Lactation increases fluid requirements by approximately 12–16% relative to non- reproducing individuals in Western populations [70]. It further increases from pregnancy (3.0 L per day) to lactation (3.8 L per day), though there is likely intra- and inter- population variability depending on lifestyle, environment, and subsistence patterns [3, 8, 29]. Although drinking more water likely does not increase milk production [72], it does prevent dehydration in the lactating individual.

Typical human milk production (86–88% water by volume) is highly variable and ranges from 550 to 850 mL per day [73]. In lactating Gambian women who partook in daily fasting during Ramadan, decreased water intake during the day resulted in altered milk synthesis, as indicated by decreased milk lactose concentration and hyperosmolality, increased milk sodium concentration, and an altered sodium to potassium ratio [74]. These results indicate changes in the integrity of the secretory cells of the mammary gland, which typically have tight junctions between adjacent cells and do not allow for paracellular transport under normal conditions after closure in the hours to days postpartum when mature milk is produced [15, 74, 75].

### Lactation and heat stress

Inquiries into heat stress and lactation are also limited by the variability underlying species differences in milk synthesis. For example constraints on lactation in rodent models have been attributed to limitations on central processes (i.e. food intake, absorption, and assimilation) and peripheral processes (i.e. those supporting the mammary gland’s synthetic capacity) [76–78]. However, both central and peripheral processes may ultimately be constrained by thermoregulatory capabilities and the need to dissipate metabolic heat [27, 79, 80]. Increased temperatures decrease lactation performance by decreasing milk energy output [81] and yield [82, 83]. In humans, maternal core temperatures increase for at least 6 months postpartum during exclusive breastfeeding [84]. However, limited research has been conducted on hyperthermia during lactation in humans.

Much of our understanding on heat stress during lactation is derived from those considered milk production animals (*cf* [85, 86]. Research on increased ambient temperature and humidity demonstrates both direct and indirect effects on dairy cattle [85, 86] that may result from a trade-off between thermoregulation and reproduction [87]. Direct effects of heat stress on lactation include alterations in macronutrient metabolism, liver function, increased incidence of mastitis, up to a 40% reduction in milk yield, and milk composition changes [85, 88, 89].

Many of these negative effects on health, metabolism, and lactation performance may also result from exposure to hyperthermic conditions during the non-lactating period and are carried-over into the subsequent lactation, even when conditions have returned to normothermic [85]. Beyond metabolic impacts, heat stress may alter hormonal profiles during lactation, including changes to somatotropin, thyroid hormones, and glucocorticoids [90, 91]. However, both rodents and dairy cattle have limitations as models for humans. These species have vastly different life histories and it is unclear how generalizable insights from laboratory rodent studies may be. Additionally, dairy cattle have been selectively bred to produce high volumes of milk in excess of a calf’s needs with known life history trade-offs[92].

## IMPLICATIONS FOR PUBLIC HEALTH AND MEDICINE

Climate change contributes to the dual burdens of heat stress and water insecurity, creating stressors during pregnancy, parturition, and postpartum. As a result, there are immediate, life course, and intergenerational consequences of chronic exposures to extreme heat and water insecurity. Pregnancy, partition, and lactation emerge as particularly vulnerable life stages that require focused support. However, the impacts of climate change are unequally distributed within and between populations, due to political economic processes. These processes can enhance vulnerabilities by constraining resources necessary for individuals to acclimatize to or mitigate the effects of climate change.

We have limited our focus to the physiological processes that increase vulnerability to heat stress and water insecurity in this review. However, it is important to note that climate change also increases risks of exposure to vector borne diseases, diarrheal illnesses, population displacement, malnutrition from crop failures or food shortages, and gender-based violence [44].

The Anthropocence, as conceptualized by Margaret Lock [93], Ayo Wahlberg, [94] and others, is characterized by multiple biological and social challenges that act to create situational, localized, and exposed biologies within and across populations. Challenges such as water insecurity and heat stress can alter the epigenome, changing gene expression in ways that may impact long-term health and well-being, including reproductive health. While stressors such as environmental pollutants have been the primary focus of these exposed/situated biologies [93, 94], chronic exposure to heat stress and water insecurity present many of the same social and physiological pressures.

Throughout this paper, we have reflected on the typical physiological trade-offs associated with pregnancy, parturition, and lactation. We have also demonstrated how these physiological processes are challenged by increased heat stress and water insecurity. Ecological trade-offs through changes to allocation, function, and the limitations of evolved biochemical pathways will shape how humans can mitigate or acclimatize to climate challenges. Understanding both the capacity for plasticity as well as the associated constraints of our species with environmental extremes are critical. However, we recognize that these biological adaptations are unlikely to provide enough flexibility to respond to the extreme climate changes we face in the coming decades. Biocultural processes are necessary to buffer the limitations of these evolved systems of temperature extremes.

The limits of physiological adaptations during these life history stages bring attention to the need for a global and robust response to mitigating climate change prioritizing those most likely to be negatively affected and with the least means to cope with these challenges. Practitioners actively recognizing and responding to these challenges can help shape interventions and reduce barriers to heat and water stressors.

### Pregnancy, water insecurity, and heat stress: implications for practitioners

Climate and water insecurity are ecological stressors forcing parents to make difficult decisions between optimal practices for survival and reducing human suffering. In addition to direct physiological mechanisms linking heat exposure to maternal and infant outcomes, several indirect or mediating factors might play a role (Fig. 1). For example, in Burkina Fasso, pregnant women reported that extreme heat interfered with medical care during pregnancy and parturition, because of heat stress and dehydration related to walking to the health clinics [95]. Additionally, women reported that cultural practices of postpartum seclusion, as well as maintenance of physical work (especially fetching and hauling household water) increased exposures to heat-related vulnerabilities [96].

Chronic heat exposure and heat stress have marked effects on maternal mental health and can lead to increased psychological stress and anxiety [97]. A large body of literature has linked maternal anxiety and stress exposure to adverse birth outcomes such as low birthweight and prematurity [98, 99] although studies of mental health effects of heat stress during pregnancy are limited [44]. Furthermore, heat stress and dehydration affect cognitive ability and health behaviors such as physical activity patterns, which might mediate or exacerbate relationships between heat stress and maternal and infant outcomes [43].

Other moderating variables might impact an individual’s experience with water stress and heat. These include pregnancy weight, chronic diseases, ethnicity, socioeconomic status, and age. For instance, underweight and lower birthweight individuals experience a higher risk of neonatal mortality, and chronic diseases like diabetes or hypertension, chronic immune diseases, impaired metabolic function, cardiovascular disease, developmental challenges, and neonatal mortality [100, 101].

Meta-analyses show that associations between reduced birthweight and high temperatures vary by ethnicity, socioeconomic status, and age. These authors found consistency in the direction of the effect even with variations in the effect size. Although there were lower impacts on some populations, these were likely driven by access to heat mediation opportunities, not biological differences in groups. The most pronounced relationship this study was found among Black, Indigenous, or Hispanic people when compared to their white peers. Associations between exposure to heat and preterm birth were strongest among people with low socioeconomic status, and among those who were under 22 or over 40 [37]. The fact that these effect sizes are smallest in areas with high use of air conditioning indicates that these differences are not driven by biological differences, but instead by health disparities among populations.

Maternal physical health or body composition might also play a moderating role. Analyses of the U.S. 2009-2012 National Health and Nutrition Examination Survey (NHANES) show that adults who are dehydrated are more likely to be obese [102], and increased water needs associated with factors such as higher body weight might put people with obesity at greater risk of dehydration in situations of heat exposure. Extreme heat exposure also interacts with cardiovascular and respiratory diseases, putting pregnant people with preexisting conditions or multimorbidity at greater risk [44].

Relatedly, previous research has illustrated that heat and water insecurity also negatively impact a wide range of food systems and subsistence practices. In addition, they are associated with a reduction in household dietary diversity and food security [103, 104]. These impacts shape infant feeding patterns during weaning and have critical impacts on child development and health across both the life course and intergenerationally [104].

### Lactation, water insecurity, and heat stress: implications for practitioners

There is a substantial literature across anthropology, global public health, and nutrition to illustrate how interactions between extreme heat and water insecurity affect human understandings, experiences, and behaviors during the postpartum transition, breastfeeding, and infant care. Edney et al. (2022) reported that the associations between heat stress and breastfeeding exclusivity varied by population with two populations showing increased breastfeeding duration and four showing decreased duration. Water insecurity has long been hypothesized to play a major role in shaping the longer breastfeeding durations of the Dobe San people, with longer lactations creating temporary hydration resources for infants and young children, who could not safely hyperhydrate as adults in the group can. Part et al. [105] working within the Dobe San communities in Burkina Faso found that increased daily temperature was associated with a decrease in breastfeeding. On the hottest days of the year, older infants nursed almost 25 minutes less than similarly aged infants on cooler days.

Infant water needs are a common concern named in studies of barriers to exclusive breastfeeding, especially in communities where chronic exposure to extreme heat and water insecurity are pervasive [104]. In these settings, newborns and infants are often given water or water-based liquids as a supplement to, or replacements for, breastfeeding when temperatures are extremely hot. Such supplementation occurs for a variety of reasons, including concerns that infants are at risk of dehydration due to the heat as well as concerns that one’s own milk supply is being negatively impacted by the heat, and therefore, increasing the infants’ risk of dehydration [105–109].

A recent study on the effects of heat on postpartum Kenyan women and infants [110] eloquently lays out the biocultural dimensions of chronically high ambient temperatures and water insecurity for maternal-infant health. It also raises additional concerns for women’s and newborn’s health at the intersection of climate and water insecurity. For example, participants spoke about newborns’ presenting with blistering skin and sores in their mouth due to exposure to extreme heat. These heat-induced conditions caused significant pain for infants while they were being held and breastfeeding and may impact frequency and duration of infant breastfeeding episodes and ultimately milk supply.

Household water insecurity among Kenyan participants in the prior study also made it difficult for postpartum women to maintain personal hygiene, and participants expressed concerns about women breastfeeding infants when mothers did not have resources to bathe themselves. Participants also emphasized the negative psychological toll of experiencing extreme heat and water insecurity postpartum. They noted that postpartum women suffered the consequences of heat and water insecurity in ways that created serious barriers for them to care for themselves and their newborns [110].

They also found that Kenyan mothers could not practice skin-to-skin contact—an evidence-based practice recommended to help promote infant thermoregulation, respiration, and breastfeeding. This was due both to extreme heat and because it was too painful for the newborns. Community members also described globally agreed upon recommendations for exclusive breastfeeding 0–6 months were described as unrealistic, citing challenges of dealing with water insecurity and extreme heat.

In households with extremely constrained resources, water may be one of the few resources people have to cope with extreme heat, both for themselves and for their infants. As discussed above, infants rely on others to co-regulate their temperature for both heating and cooling. Damp cloth baths, which are often used to reduce fevers, also help cool infants on hot days. When water is scarce, however, it may be difficult to prioritize bathing over other activities, such as preparing food and drinking water [5, 9]. Morbidity and mortality rates of newborns and young infants who are not exclusively breastfed in these resource constrained settings are substantially higher than exclusively breastfed infants [12, 111]. These findings elucidate the everyday lived experiences and suffering that women and infants are facing as a result of climate change-related heat exposure and water insecurity, which have not yet been widely discussed in the literature [112–114].

### Call for research integrating reproduction, climate, evolutionary medicine, and global public health

Due to the rapidly worsening climate crisis, we are entering a cultural and evolutionary crossroads as pregnancy rates and the average heat index increase continue to climb and global water security decreases [14]. What emerges when taking this into account with our evolutionary and physiological constraints presented here is that chronic exposure to extreme temperatures along with chronic experiences of water insecurity is challenging at best—and deadly at worst.

Evolutionary medicine can provide the critical work of addressing these issues in a cohesive framework. By emphasizing this framework, researchers and practitioners would be further empowered to make strong predictions on how human populations will be impacted by exposure to rapidly changing environmental conditions. Research integrating climate, evolutionary medicine, and global public health is a critical priority.

Evolutionary theory generates critical questions that remain unanswered regarding the effects of climate change on human reproductive health. There are fundamental questions that would aid practitioners and disaster planners in identifying other potential risk factors. For instance, there are differences in reproductive outcomes between populations that are well-adapted or acclimatized to extreme heat or extreme variability in water security? How do these population’s reproductive and pregnancy outcomes compare to those who experience exposures to extreme climate events more sporadically or relatively recently?

Evolutionary medicine researchers could also empower practitioners by determining what kinds of epigenetic changes are being triggered in the developing fetus or embryo? How might these result in intergenerational effects of climate disasters? What trade-offs may be occurring as part of maternal-offspring conflict surrounding hyperthermia and dehydration in the pregnant or lactating individual? How would this potential pressure influence relatively adverse (or beneficial) outcomes for pregnant people relative to their gestating offspring or infants? How do these tradeoffs shift over the course of gestation, lactation, and infancy?

**Figure 2.**
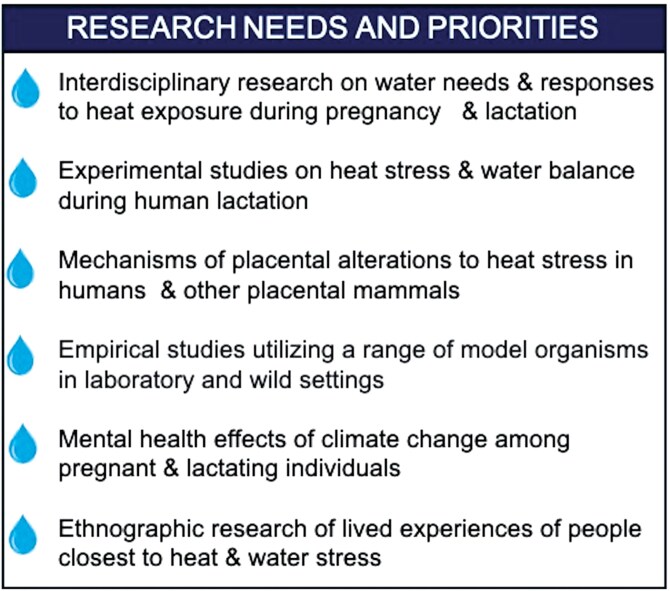
Research needs and priorities regarding water and heat stress on pregnancy and lactation.

An interdisciplinary research approach informed by evolutionary perspectives, are critical to ascertaining the diversity of human water needs and responses to exposures to extreme heat during pregnancy, lactation, and infancy (Fig. 2). Additional research applying evolutionary medicine to secondary outcomes of climate driven heat stress and water insecurity would be a powerful addition. For instance, assessing the effects of climate change and its impacts on population health through climate disasters, increase exposure to conflict and violence, food insecurity, prenatal stress, and the intergenerational impacts of these on pregnancy and lactation is critical to expand our understanding [115, 116]. Experimental studies, particularly those that utilize an ecological and evolutionary framework, are necessary to fill gaps in understanding the mechanisms of heat stress and water balance during pregnancy, human lactation, and infant feeding. This includes some basic human biology and reproductive ecology research.

Human variation in some groups reflect physiological mechanisms that enable increased adaptation to hot and/or arid climates, however, ambient temperatures are likely to outpace these adaptations. This means that reaction norms will no longer be adaptive, as phenotypic plasticity is still constrained by an upper critical temperature [117–120]. However, this critical temperature is still not fully known. The theoretical upper critical limit for human thermoregulatory adaptability is estimated to be a wet bulb temperature of 35°C. This estimate may be inaccurate and until recently was not empirically explored [118]. It is unclear how this upper critical temperature limit is impacted by reproductive status in humans. It is also unclear what the effects of approaching that limit may be on both the developing neonate and maternal health. Investigations incorporating a combination of methods to estimate how this limit may change over the course of reproduction would be a powerful addition to our understanding of these limits.

Building a basic understanding of human lactation is also needed. For example, it is not known what the ideal micronutrient composition of milk is for humans, which factors influence maternal transmission and/or infant absorption and use, how the local environment may alter micronutrients in milk, or how milk volume impacts nutrient composition (i.e. are micronutrients concentrated when milk is produced in lower volumes or do lower volumes of milk mean lower micronutrients) [121]. Similarly, it is not known exactly how physiological stressors, such as nutrient/energetic, thermal, or water stress, impact maternal glucocorticoids in circulation and milk, nor has it yet been fully explored how the developing neonate responds to the vertical transmission of maternal hormones [122, 123], which may have intergenerational effects on lactation performance of offspring [124].

It is unclear how hormones such as cortisol may confer a predictive adaptive response [125] in some environments, which may afford some advantage within the scope of the reaction norm. Thus, we suggest that (i) investigations on the composition of milk should include some measure of milk volume so that patterns can emerge and (ii) there is a great need for understanding lactocrine programming [126] within the context of climate change. Interdisciplinary research and evolutionary theory and methods of biological anthropology are necessary complements to current efforts to understand intersections of climate and human health.

However, ethnographically informed evolutionary medicine research is also vital to this work. Future research on climate and human reproduction are enriched when they are grounded in the lived experiences, local cultural and ecological knowledge, and expertise of populations most impacted by heat and water stress [68]. Many of the populations consistently impacted by heat waves and water insecurity are in low-income countries, indicating the critical need to expand studies outside middle- and high-income areas including local researchers.

Evolutionary perspectives in medicine and public health strengthen education, counseling, and support of pregnant, postpartum, and lactating people in this global climate crisis. There are many examples of how evolutionary perspectives are embedded into practices that improve the delivery of care throughout the perinatal and postpartum period—including group prenatal counseling, ensuring women are supported during labor, delivery, and immediately postpartum by a skilled birth attendant or other support person (e.g. *doula*), and integrating mental health and psychosocial support during one’s physical and psychological transition to parenthood. Communities can be empowered to develop stronger mutual-aid, disaster preparedness, response, and resilience resources, which ensure that the unique needs of special populations—including pregnant and lactating people—are prioritized appropriately.

Similarly, adaptation and mitigation of climate change and water insecurity require a strong understanding of the context-specific nature of human–environment interactions that shape human reproduction. Maternity-specific cooling areas, shelters, and water sharing programs may simultaneously serve as relief from heat and water stress and sites where education, counseling, and services may be delivered to those in need. The effective translation and application of evolutionary perspectives into interventions that support healthier pregnancies and birth and breastfeeding outcomes will require integration of cultural, ecological, and political economic dimensions of sexual and reproductive health. For example, information about the risks of heat exposure and water insecurity, along with competencies for how to mobilize existing resources for context-specific interventions, should be incorporated into practitioner education. Timely interventions for adaptation and mitigation globally, including those derived from convergence frameworks [127], must include ethical engagement with local communities and Indigenous knowledges, and must be oriented to intervene on the unique vulnerabilities of childbearing people, infants, and young children.

## Conclusion

Pregnant and lactating people emerge as a particularly critical demographic to focus on when trying to understand the local and global impacts of climate change on human health across the life course and intergenerationally. We have explored how rising global temperatures and concurrent water insecurity exacerbate physiological challenges during pregnancy and lactation—stages already marked by heightened metabolic and hydration needs. These individuals are uniquely vulnerable to heat stress and dehydration due to their increased basal metabolic rates and demands for thermoregulation and hydration. As temperatures rise and water availability diminishes, these physiological demands become harder to meet, posing significant risks to maternal health and fetal development. The repercussions extend beyond immediate health effects, influencing birth outcomes, infant growth, and long-term, and intergenerational health trajectories.

From an evolutionary perspective, humans have adapted over millennia to manage environmental stressors. However, rapid climate change outpaces our adaptive capacities, forcing individuals to contend with increasingly severe and unpredictable conditions. Evolutionary trade-offs that once conferred advantages may now heighten vulnerabilities, underscoring the dynamic interplay between biological adaptations and contemporary environmental challenges. A deeper and nuanced understanding of human biological variability and the impacts of climate crises on human reproduction can generate novel, and essential, insights to population-specific needs for the highly contextual impacts of climate change. Evolutionary theories and methods should be integral to current scientific efforts that link climate change and health globally.

Effective interventions must address both the direct physiological impacts of heat stress and water insecurity and the broader socioeconomic and cultural factors that mediate these effects. This includes improving access to adequate healthcare, clean water, nutrition, and ensuring appropriate shelter. Also critical is enhancing healthcare infrastructure to monitor and mitigate heat-related health risks, integrating climate resilience into maternal health programs, and promoting sustainable water management practices. Although this is a systemic not individual challenge, prioritizing the empowerment of individuals with knowledge about heat stress management, hydration strategies, and adaptive behaviors during pregnancy and lactation can support lifesaving self-care practices.

Addressing the intersection of climate change, pregnancy, and lactation requires a multifaceted approach that integrates evolutionary insights with robust public health strategies. Collaborative efforts among policymakers, healthcare providers, researchers, and communities are essential to develop and implement effective strategies that protect maternal and infant health in the face of climate change. Understanding the adaptive responses shaped by evolutionary processes and implementing evidence-based interventions can mitigate the adverse effects of climate change on maternal and infant health, promoting health equity and resilience across diverse populations globally[128].
